# Comprehensive Wellbore Stability Modeling by Integrating Poroelastic, Thermal, and Chemical Effects with Advanced Numerical Techniques

**DOI:** 10.1021/acsomega.4c09013

**Published:** 2024-12-18

**Authors:** Eissa M. Shokir, Samy Sallam, Mostafa M. Abdelhafiz

**Affiliations:** † Department of Petroleum Engineering, Cairo University, 12613 Giza, Egypt; ‡ Institute for Disposal Research, TU Clausthal, 38678 Clausthal-Zellerfeld, Germany; § Faculty of Engineering and Technology, Future University in Egypt, 11835 Cairo, Egypt

## Abstract

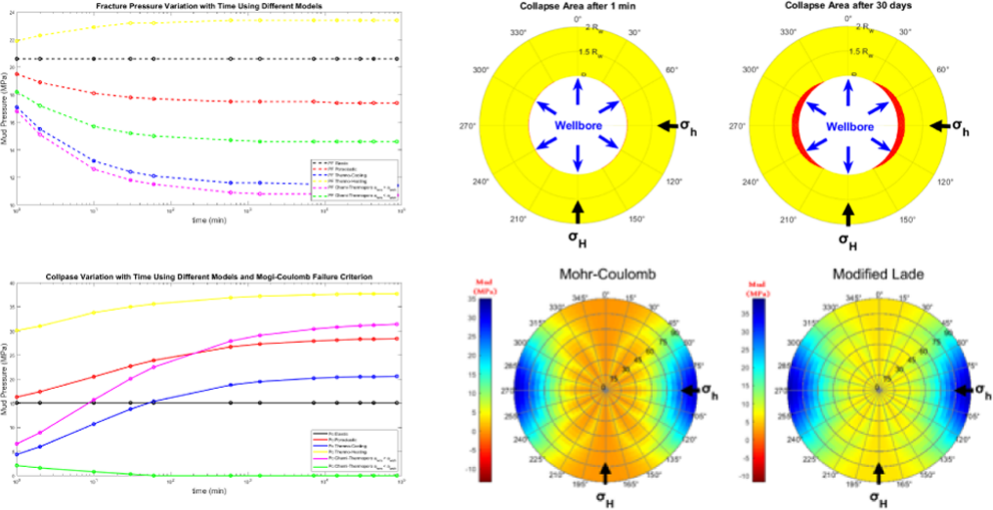

Wellbore stability in extreme drilling environments remains a critical challenge. This study advances the understanding of these complexities through a comprehensive numerical modeling approach. By incorporating thermal, chemical, and hydraulic effects, four refined models were developed to simulate wellbore behavior under high pressures and temperatures. A comparative analysis of four failure criteria and a detailed investigation into the impact of fluid properties on pore pressure and stress distribution provide novel insights. The results indicate that pressure distribution and stress variations around the wellbore are significantly influenced by poroelastic, thermal, and chemical effects. The poroelastic effect increases pressure due to overbalanced drilling conditions, while thermal effects vary with fluid temperature, leading to notable pressure changes. Chemical effects are significant, with lower salinity mud increasing pore pressure and higher salinity decreasing it. Thermal effects primarily dominate stress distribution, altering radial, tangential, and axial stresses, with tangential stresses peaking in the direction of minimum horizontal stress. Collapse area predictions suggest that Mohr-Coulomb and Drucker-Prager criteria predict larger collapse areas compared to Mogi-Coulomb and Modified-Lade criteria, indicating a more conservative approach. Poroelastic effects slightly enlarge collapse areas due to increased pore pressure, while thermal effects reduce collapse areas with cooling and increase them with heating. Higher salinity mud improves formation stability by enhancing effective stress and reducing pore pressure. The results demonstrate that using higher salinity mud enhances formation stability and that careful management of temperature can mitigate stress variations and collapse risks. Regular monitoring and adjustments based on wellbore stability models are essential for optimizing performance and safety in drilling operations. The findings reveal that optimizing mud salinity and carefully managing temperature can effectively enhance formation stability, which offers practical guidelines for mitigating wellbore risks and optimizing drilling operations in challenging formations.

## 1 Introduction

Wellbore instability is one of the most challenging problems in the oil and gas industry and is the reason for most drilling difficulties. It is estimated to have caused significant annual global losses, and 90% of these problems occur in low-permeability shale, which represents 75% of all drilled formations.^1^ Lately, the proven oil and gas resources discovered in deep-water fields have boomed progressively, and the need for the recovery of these petroleum reserves makes it imperative to drill in extremely harsh environments, where the pressure and temperature are very high, and the formation is chemically active. To face this major challenge, it is essential to accurately consider all the factors affecting wellbore stability, including stresses, pressure, temperature, and chemical effects. To understand how the poroelastic, thermal, and chemical effects influence wellbore stability, one should understand how these effects influence the pressure, temperature, and stress distributions around the wellbore and how they are interdependent. Starting with the poroelastic effect, drilling using mud with a different pressure than the formation pressure causes fluid diffusion between the formation and the wellbore, which in turn changes the stresses and pressure around the wellbore. Moving to the thermal effect, drilling mud has a different temperature than the surrounding rock, which changes continuously by contact with the formation during circulation. This temperature change causes heat transfer between the wellbore and the formation by conduction and convection cooling the rock at larger depths and heating it at shallower ones.^2^ Heat transfer has two impacts on wellbore stability. First, the stress profile surrounding the wellbore is changed by the generated thermal stresses. Second, the distribution of pore pressure is impacted by temperature fluctuations. Finally, the chemical effect is caused by the difference in salinity between the drilling mud and the formation fluid. This salinity difference causes water and salts to transfer between the wellbore and the formation. This affects wellbore stability by changing the pressure and stress distribution and reducing shale strength around the wellbore.

The first attempt to study wellbore stability was by using a time-independent linear elastic model to calculate the concentrated stresses around the wellbore and compare them with rock strength using proper failure criterion.^3−5^ The poroelastic theory was first developed by Biot^6^ and was further developed by Detournay and Cheng,^7^ who studied the poroelastic effects on delayed borehole instability and shear failure initiation inside the rock.^7^ Cui et al.^8^ also developed a time-dependent poroelastic model for inclined boreholes using a loading decomposition scheme.^8,9^ Palciauskas and Domenico^10^ first introduced the thermoporoelastic theory by studying the mechanical response of rock to heating during nuclear waste storage. It was further developed and several studies conducted wellbore stability analysis based on linear thermoporoelastic models neglecting convective heat transfer.^11−13^ Roohi et al.^14^ used a linear thermoporoelastic model to estimate the optimum reamer/bit size ratio in reaming while drilling (RWD) technology. The assumption of neglecting the convection heat transfer in mid or high-permeability formations is not valid. Therefore, Wang and Dusseault^15^ consider this convection effect in their thermoporoelastic model for steam injection in high permeability formation. Chen and Ewy^16^ studied both conductive and convective heat transfer for both a permeable and an impermeable boundary. Also, a fully coupled conductive-convective thermoporoelastic model during drilling in high-permeability sandstone was developed by Farahani et al.,^2^ and Gomar et al.^17^ Thermal osmosis and thermal filtration effects were also considered in some studies such as Zhou et al.,^18^ Gao et al.,^19^ Liu et al.,^20^ and more recently, Fan and Jin^21,22^ have studied the poroelastic and thermal convective effects considering the shale as a semipermeable boundary for nonhydrostatic in situ stress conditions. Some researchers have considered the chemical effect in analyzing wellbore stability taking into account thermal stresses and the flux of both water and solutes from drilling fluids into and out of shale formations.^23−25^ Chen and Ewy^26^ have used a chemo-poroelastic model to calculate pressure, stresses, and critical mud weights with and without including the undrained loading effect. Chen et al.^1^ have studied the effects of mechanical forces and poroelasticity, as well as chemical and thermal effects on shale behavior. The effect of shale hydration on strength reduction has been studied by many researchers using different drilling fluids and shale samples at different soaking times.^27−29^ Additionally, some studies have considered the impact of other factors on wellbore stability such as the presence of fractures,^30^ rock strength anisotropy,^31^ and the anisotropy of hydraulic and thermal conductivity of the rock formation.^32^

Additionally, several research efforts have been invested in order to evaluate the mechanical, chemical, and thermal effects on wellbore stability,^33^ while investigating the effect of different failure criteria as by Aslannezhad et al.^34,35^ They investigated the effect of variation in temperature, mud salinity, and cohesion on the determination of a safe mud window. In the solution of their model, they used the complementary error function approach to describe transient phenomena of the temperature and pressure. Although this approach is widely used to obtain an analytical solution to the problem, it is primarily useful for short-term, transient analysis.

While significant progress has been made in understanding and modeling wellbore stability under complex conditions, a comprehensive and integrated approach that simultaneously considers the coupled effects of poroelasticity, thermal, and chemical processes, as well as the influence of different failure criteria on wellbore stability in deep, high-pressure, high-temperature environments remains limited. This paper investigates the individual and coupled effects of poroelasticity, thermal, and chemical processes on wellbore stability in deep, high-pressure, high-temperature environments. The paper also assesses the influence of different failure criteria on wellbore collapse predictions. From that extent, four numerical models are developed to calculate the stresses acting on the wellbore according to the individual effects of poroelasticity, thermal, and chemical processes. The coupled interactions between poroelastic, thermal, and chemical effects on wellbore stress and pressure distributions are investigated. Additionally, the performance of four failure criteria in predicting wellbore collapse under various loading conditions and environmental factors is evaluated. The paper is divided into four main sections. In the first section, a brief introduction and review of the literature body is highlighted. In Section 2, a description of the model development process is presented. In this section, the mathematical description of the four models is elaborated. In Section 3, the results of the models described are presented, in addition to a discussion of the results. The validation of the models is also presented. Finally, the conclusions and recommendations are highlighted in Section 4.

## 2 Methods

Modeling wellbore stability involves five key steps. First, the in situ principal stresses are converted into the wellbore coordinate system. Second, the distribution of temperature, pressure, and stresses around the wellbore is computed, including various models (Elastic, Poroelastic, Thermoporoelastic, and Chemi-Thermoporoelastic). Third, the three principal stresses at each point around the wellbore are determined. Fourth, a failure criterion is applied to assess whether the wellbore can sustain the applied stresses or if failure is imminent. These criteria help predict the potential collapse area around the wellbore for a given mud weight. Finally, the computed principal stresses are compared to rock strength using the applied failure criterion to establish the safe mud window and optimal wellbore trajectory for drilling. These steps will be explained in more detail in the following subsection.

### 2.1 Stress Transformation

**Figure 1:**
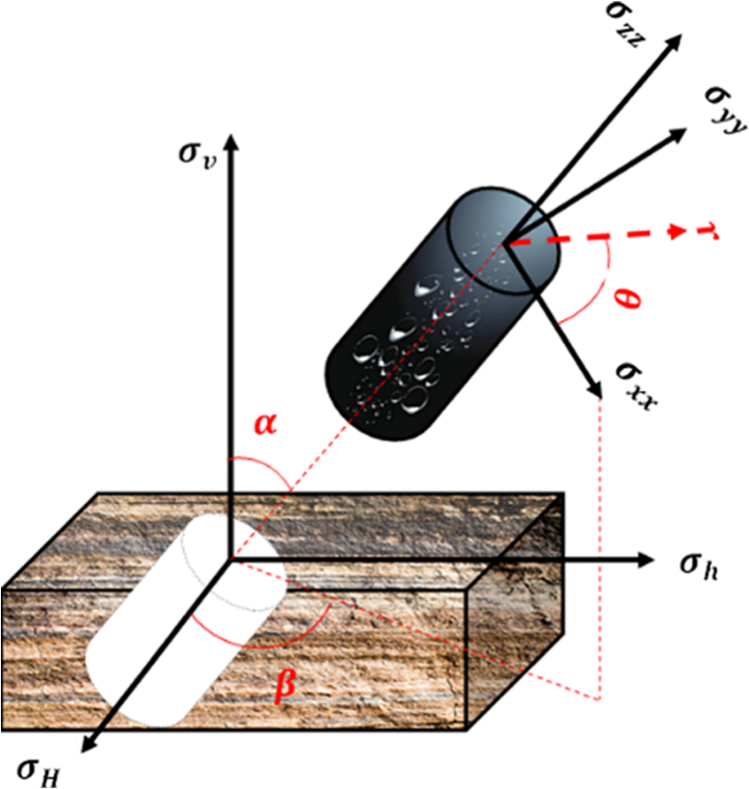
Conversion of stresses between different coordinates systems.

The in situ principal stresses (σ*_v_*, σ*_H_*, σ*_h_*) representing vertical, maximum horizontal, and minimum horizontal stresses respectively, are transferred into the local wellbore coordinate system (σ_*xx*_, σ_*yy*_, σ_*zz*_) with its coinciding with the borehole axis at any azimuth and inclination angle (β, α) using the following equations from Abousleiman et al.^36^ as shown in Figure 1.
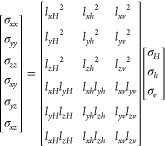
1where,
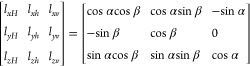
2

### 2.2 Modeling the Wellbore Stresses

The development of a comprehensive model to analyze the stress distribution around a wellbore, considering various influencing factors such as hydraulic, thermal, and chemical effects are outlined. We begin by discussing the foundational linear elastic model based on Kirsch’s solutions, which serves as the starting point for our analysis. Kirsch’s solutions describe the stress distribution around a circular hole in an infinite, homogeneous, isotropic elastic medium, providing the basis for understanding the wellbore stress response under different conditions. Building on this, poroelastic effects to account for fluid pressure interactions within the formation are incorporated, followed by thermoporoelastic effects to include thermal-induced stresses. Finally, we extend the model to chemo-thermoporoelasticity, capturing the combined influence of chemical reactions, temperature changes, and fluid pressure on the stress state around the wellbore.

#### 2.2.1 Governing Equations

Starting from the linear elastic model, which assumes that the concentrated stresses around the wellbore only result from removing the rock column during drilling ignoring the hydraulic, thermal, and chemical effects. These stresses can be calculated using Kirsch’s solutions^37^ as in (eqs 3–8).
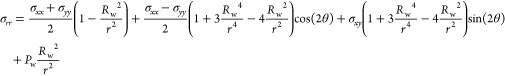
3
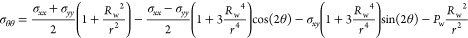
4
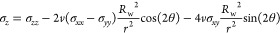
5
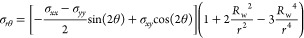
6
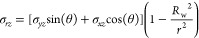
7
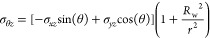
8where the subscripts (*rr*, θθ, and *z*) denote the stresses in cylindrical coordinates in the radial, tangential, and axial directions, respectively. The terms σ_*r*θ_, σ_*rz*_, and σ_θ*z*_ represent shear stress components in the radial-tangential, radial-axial, and tangential-axial planes, respectively. σ_*xx*_, σ_*yy*_, and σ_*zz*_ refer to the far-field principal stresses in the Cartesian coordinate system, aligned with the *x*, *y*, and *z* directions, while σ_*xy*_, σ_*xz*_, and σ_*yz*_ denote shear stresses. *R*_w_ is the radius of the wellbore, *r* is the radial distance from the wellbore center where stresses are evaluated. The angle θ is measured in the cylindrical coordinate system from a reference direction. *P*_w_ is the wellbore pressure, and Finally, *v* denotes the Poisson’s ratio of the rock.

The hydraulic diffusion effect is addressed through the poroelastic model, which accounts for the changes in pressure and stresses resulting from the fluid exchange between the wellbore and the surrounding formation. This exchange is driven by the pressure differential between the drilling mud and the formation’s pore pressure. based on Biot’s theory,^6^ the transient hydraulic diffusion can be given by eq 9.
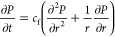
9where *P* is the pore pressure, *t* is the time. *c*_f_ is the diffusivity coefficient for fluid flow in the porous medium. The coupling between the stress–strain relationship and the hydraulic diffusion is done through the constitutive equations for a poroelastic medium as by the following equation.
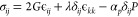
10Here, *G* is the shear modulus of the rock, and ϵ_*ij*_ is the strain tensor component. λ is the Lamé’s first parameter δ_*ij*_ is the Kronecker delta, which is 1 when *i* = *j* and 0 otherwise, ensuring that *λδ*_*ij*_ϵ_*kk*_ only affects the normal components of the stress tensor, and α_p_ is the Biot’s coefficient.

The thermal effects are considered by coupling the transient temperature variation with the hydraulic formation pressure and the induced thermal stresses due to the expansion/contraction of the rock grains. The transient temperature distribution is given eq 11.
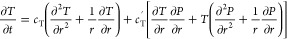
11where *T* is the formation temperature, *c*_T_ is the thermal diffusivity of the rock. The left-hand side of the equation represents the transient heat accumulation. The first term on the right-hand side represents the heat transfer by diffusion, and the second term on the right-hand side represents the heat transfer by convection. For low permeability formations as the case in shale, this last term can be neglected,^38^ and eq 11 becomes,
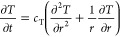
12

Therefore, eq 9 can be rewritten to account for the pressure/temperature coupling following.
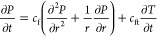
13with *c*_ft_ as a coupling coefficient that links temperature change to pore pressure. Finally, the chemical effect is considered by the chemi-thermoporoelastic model, which incorporates the changes in pressure, stresses, and shale strength due to the transfer of water and salts between the wellbore and the formation, driven by salinity differences. The pressure change is quantified using the following equation.^1^
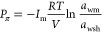
14where *P*_π_ represents the osmotic pressure, *R* is the universal gas constant, *T* is the formation temperature, *V* is the partial molar volume of the water, *I*_m_ is the shale membrane efficiency, and *a*_wm_ and *a*_wsh_ denote the water activity of the drilling mud and shale, respectively. Research has extensively examined the water activity and membrane efficiency of shale, along with the factors influencing them.^27,39,40^ Generally, higher fluid salinity results in lower water activity. Conversely, lower membrane efficiency allows ions to move more freely between the mud and the shale, reducing osmotic diffusion.

This proposed model captures the transient nature of pore pressure and temperature distributions through pore pressure variation, temperature variation, and chemical instabilities components. Although the base model for linear elastic solution considers a steady-state condition, eqs 9 and 12 describe the time-dependent evolution of pore pressure and temperature, respectively, considering the effects of fluid diffusion and thermal conductivity in the formation. Furthermore, eq 13 introduces a coupled temperature–pressure effect, accounting for temperature-induced changes in pore pressure. This approach allows for dynamic stress redistribution around the wellbore as temperature and pore pressure evolve over time. By incorporating these transient effects, the model provides a more comprehensive and realistic analysis of wellbore stability.

#### 2.2.2 Numerical Solution

In this section, the numerical methods employed to solve the governing equations for stress calculations around the wellbore are outlined. The finite difference method with forward approximation is utilized to discretize and solve these equations, and four distinct models are defined; the linear elastic model, the poroelastic model, the thermoporoelastic model, and the chemi-thermoporoelastic model.

First, for the linear elastic model, the stresses are directly computed using Kirsch’s solutions as presented in (eqs 3–8). These solutions provide analytical expressions for the stress components around a circular wellbore in an infinite elastic medium.

Second, in the poroelastic model, the finite difference method is employed to discretize both the pressure equation (eq 9) and the stress–strain relationship (eq 10) as follows.
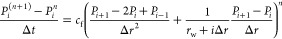
15

The hydraulic-induced stresses expressed by the following relationship.
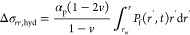
16
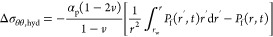
17
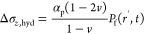
18where *P*_f_(*r*, *t*) = *P*(*r*, *t*) – *P*_o_. The total stress is then computed by adding the hydraulic-induced stresses to the mechanical stresses from the linear elastic model.

Third is the thermoporoelastic model, which extends the poroelastic model by including thermal effects. We solve the transient temperature distribution equation (eq 12).
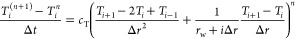
19The coupled pressure/temperature equation (eq 13) is given by the following.
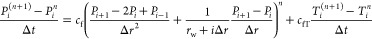
20Similarly to the poroelastic model, the induced thermal stresses are calculated according to the following equations.
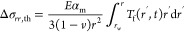
21
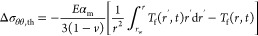
22
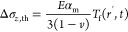
23where *T*_f_(*r*, *t*) = *T*(*r*, *t*) – *T*_o_. For this model, the induced thermal stresses shown by (eqs 21–23) and the induced hydraulic stresses shown by (eqs 16–18) are added to the linear elastic model. However, for that model, eq 20 is used for calculating the formation pressure.

#### 2.2.3 Initial and Boundary Conditions

The initial conditions for the pressure and temperature are expressed as,
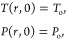
and the boundary conditions are,
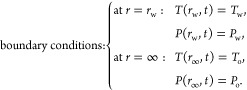


Finally, The chemi-thermoporoelastic model incorporates chemical effects by accounting for osmotic pressure changes. The osmotic pressure is computed using eq 14. This effect influences the interface between the wellbore and the drilling fluid. It is worth noting that in this work, the osmotic pressure is assumed to be steady-state, as the difference in the salinity between the formation and the drilling fluid is significant, which leads made the effect occurring instantaneously. In that case, the boundary conditions of the pressure at the wellbore wall become as follows.
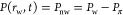
24

### 2.3 Principal Stresses Calculation

**Figure 2:**
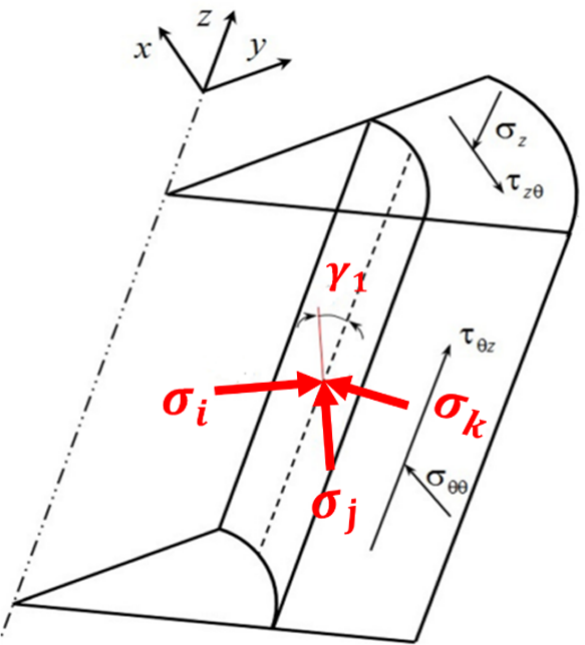
Principal stresses acting around the wellbore.

As shown in Figure 2, the radial stress is always one of the three principal stresses acting on the wellbore, and the θ–*z* plane contains the other two principal stresses, which can be calculated by the following equations.^41^
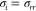
25
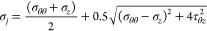
26
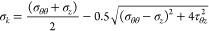
27where σ_*j*_ and σ_*k*_ are oriented at angles γ_1_ and γ_2_ from the *z*-axis of the wellbore, respectively, and can be calculated by the following equations.
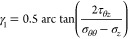
28
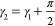
29

### 2.4 Failure Criterion

To assess the stability of the rocks surrounding the wellbore, a failure criterion should be assigned. A rock fails when the surrounding stress exceeds its tensile or shear strength, whichever is reached first, and the type of failure depends on rock lithology and the applied stress. Table 1 presents a comprehensive overview of various rock failure criteria, detailing the governing equations, relevant rock parameters, linearity, and the effect of intermediate principal stress (σ_2_). The table presents 5 shear failure criteria beginning with the Mohr-Coulomb criterion, followed by the Drucker-Prager criterion, the Mogi-Coulomb criterion, the Modified-Lade criterion, and finally Hoek-Brown criterion. Each criterion offers a different approach to modeling rock failure: the Mohr-Coulomb and Mogi-Coulomb criteria assume linearity and either ignore or consider σ_2_, while the Drucker-Prager, Modified-Lade, and Hoek-Brown criteria are nonlinear, with varying considerations for σ_2_.^31,37^ Additionally, the study includes the tensile failure criterion, which addresses conditions where tensile stress leads to failure. This criterion is simply comparing the minimum principle stress with the rock tensile strength. The criterion assumes a tensile failure of the rock takes place if the minimum effective principle (σ_3_^′^) stress acting on the rock exceeds the tensile strength.

**Table 1:** Rock Failure Criteria

failure mode	failure criteria	governing equationsand failure index	rock parameters	linearity	effect of σ_2_
shear failure	Mohr-Coulomb	σ_1_^′^ = UCS + *qσ*_3_^′^		linear	ignored
		FI = UCS + *qσ*_3_^′^ – σ_1_^′^			
shear failure	Drucker-Prager			nonlinear	considered
		*I*_1_^′^ = σ_1_^′^ + σ_2_^′^ + σ_3_^′^			
					
					
shear failure	Mogi-Coulomb	τ_oct_ = *a* + *b σ*_m,2_		linear	considered
					
					
		FI = *a* + *b σ*_m,2_ – τ_*oct*_			
shear failure	Modified-Lade			nonlinear	considered
		*I*_1_^″^ = σ_1_^′^ + *S* + σ_2_^′^ + *S* + σ_3_^′^ + *S*			
		*I*_3_^″^ = (σ_1_^′^ + *S*) (σ_2_^′^ + *S*) (σ_3_^′^ + *S*)			

shear failure	Hoek-Brown			nonlinear	ignored
tensile failure	tensile failure criterion	σ_3_^′^ = −St		linear	ignored
		FI = σ_3_^′^ + St			

**Figure 3:**
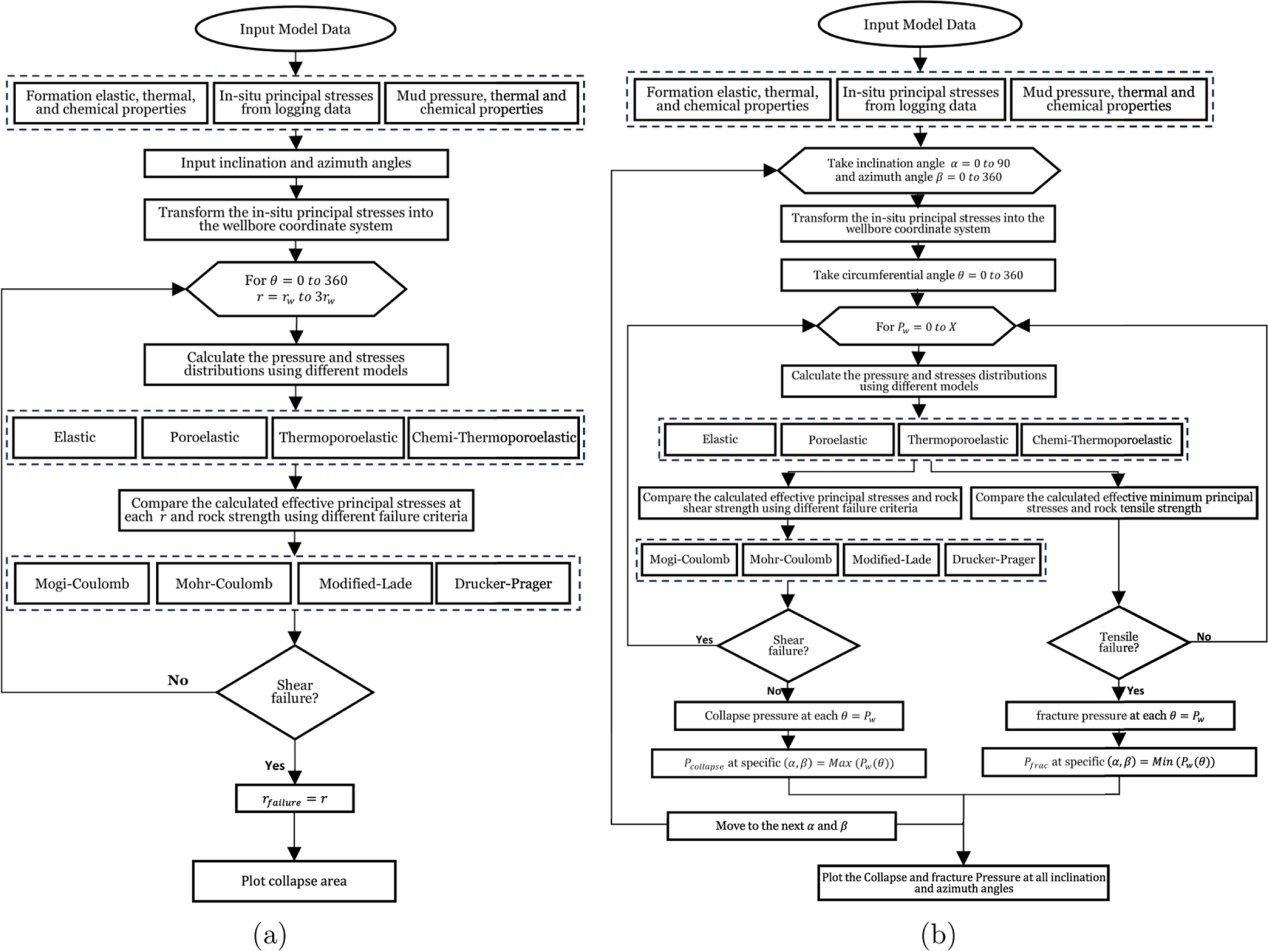
MATLAB algorithms for wellbore stability analysis. (a) Algorithm for predicting collapse areas based on rock failure criteria and input parameters. (b) Algorithm for calculating the safe mud window to ensure wellbore stability.

A MATLAB code is developed that contains the set of equations described previously, in addition to the failure criteria detailed in Table 1, which aims to predict the collapse failure according to the input parameters and also set the safe mud window. This code integrates various rock failure criteria allowing for comprehensive stability assessment of wellbore rocks. The code evaluates the input parameters to determine the likelihood of tensile or shear failure. By identifying these failure points, the code helps in defining the safe mud weight window necessary to maintain wellbore stability. The algorithm that the code follows is presented in Figure 3, illustrating the logical flow. The code first starts by importing the required input data which are formation elastic, thermal and chemical properties, and the in situ principal stresses. These data can be determined from logging data, and the mud pressure thermal and chemical properties. Second, at any inclination and azimuth angle, the in situ principal stresses have been transformed into the wellbore coordinate system. Then the pressure and stress distributions have been calculated using the different models (elastic, poroelastic, thermoporoelastic, chemi-thermoporoelastic) models. Then the calculated concentrated normal and shear stresses have been transformed into the three principal stresses acting at each point around the wellbore. Finally, the calculated effective principal stresses have been compared with the rock shear and tensile strength using the different shear and tensile failure criteria to predict the collapse area at any specific mud weight and the mud window at any inclination and azimuth angle.

## 3 Results and Discussions

### 3.1 Numerical Solution Verification

The numerical solution developed in the previous section is verified by comparing it with the models presented by Ding et al.^32^ for wellbore stability analysis, which accounts for the effects of anisotropic thermal and hydraulic conductivity. In their work, they introduced two models, first, is a semianalytical solution that uses the Stehfest method for Laplace inversion, referred to in this work as the (Laplace inversion method). The second is an analytical solution that assumes early time and small radial distance conditions, which will be referred to as the (Error function method).

**Figure 4:**
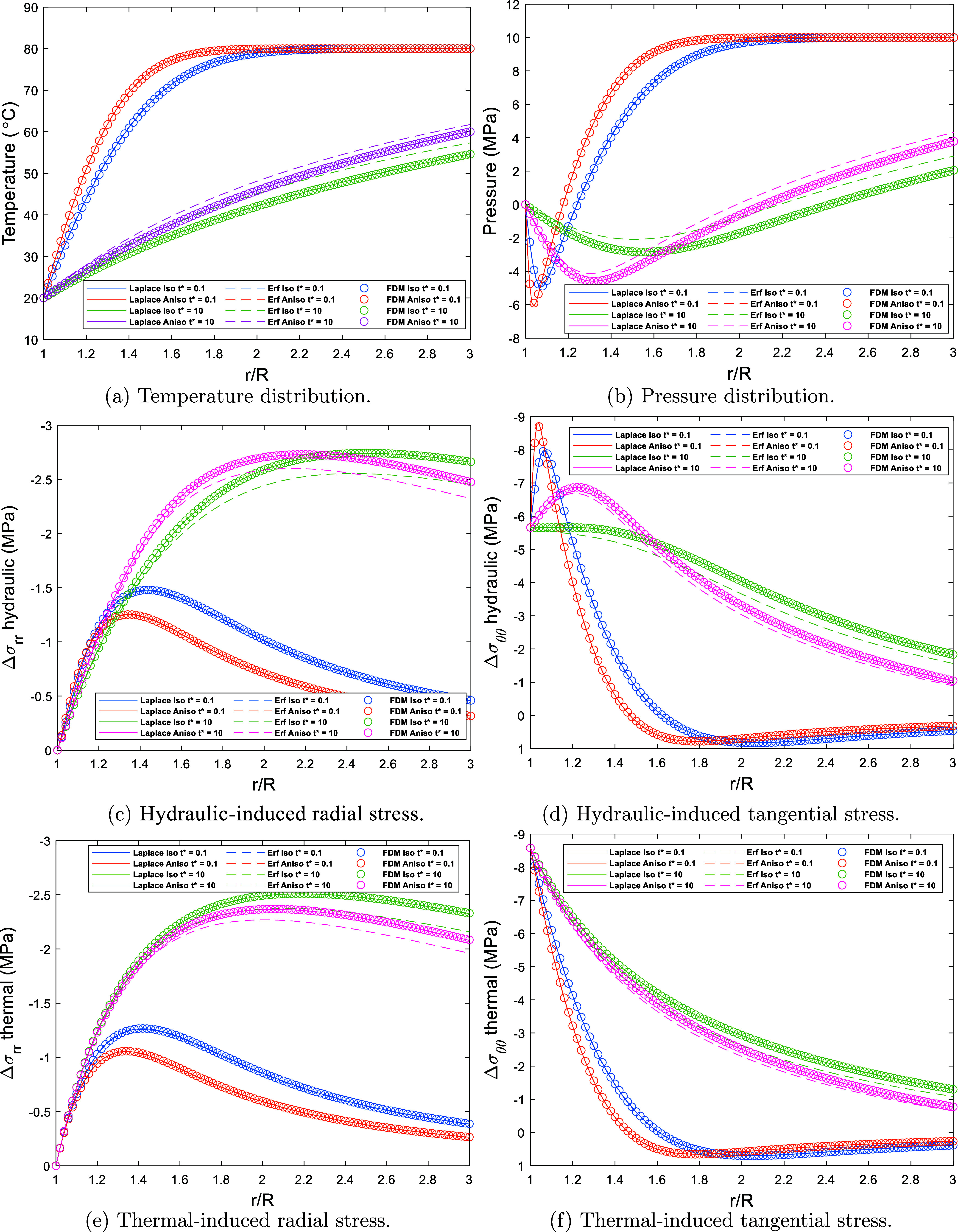
Wellbore stresses analysis results from present work at (θ = 0°), and (t* = 0.1 and 10). (a) Temperature distribution, (b) pressure distribution, (c, d) induced hydraulic stresses, and (e, f) induced thermal stresses in comparison with the models from Ding et al.^32^ (Laplace Inversion Method and Error Function Method) and the present work.

The verification results using the input data from Ding et al.,^32^ shown in Table 2 are presented in Figure 4. The figure shows the results from the present model in comparison with the models from Ding et al.^32^ according to the temperature distribution (Figure 4a), pressure distribution (Figure 4b), induced hydraulic stresses (Figure 4c,d), and induced thermal stresses (Figure 4e,4f).

**Table 2:** Model Input Parameters^32^

parameter	value
in situ principal stresses and formation properties	
overburden stress σ*_v_* (MPa)	30
maximum horizontal stress σ_*H*_ (MPa)	30
minimum horizontal stress σ_*h*_ (MPa)	20
Poisson’s ratio ν	0.3
Biot’s effective stress coefficient α	0.99
Young’s modulus *E* (MPa)	3336
hydraulic diffusivity along beddings *c*_f,∥_	7.15 × 10^–9^
hydraulic diffusivity perpendicular to beddings *c*_f,⊥_ (m^2^/s)	1.43 × 10^–9^
thermal diffusivity along beddings *c*_T,∥_	7.15 × 10^–7^
thermal diffusivity perpendicular to beddings *c*_T,⊥_ (m^2^/s)	3.575 × 10^–7^
coupled thermal-fluid pressure coefficient *c*_ft_ (MPa/°C)	0.31
volumetric thermal expansion coefficient of the formation α_m_ (°C^–1^)	9 × 10^–5^
rock matrix cohesion *c*_m_ (MPa)	5
rock matrix friction angle ϕ_m_ (°)	30
drilling data	
original pore pressure *P*_o_ (MPa)	10
original formation temperature *T*_o_ (°C)	80
borehole pressure *P*_w_ (MPa)	0
borehole temperature *T*_w_ (°C)	20
well profile data	
well radius *R* (m)	0.1
well Azimuth from σ_h_ direction β (°)	0
well inclination α (°)	90
numerical solution parameters	
time step Δ*t* (s)	1
radial distance step Δ*r* (m)	0.002
t*	
r*	*r*/*R*

The comparison is carried out by considering both isotropic (ICC) and anisotropic (ACC) conditions at two distinct times, representing short-term and long-term behavior (t* = 0.1 and t* = 10). Here, the dimensionless time t* is defined as .

For the anisotropic cases (ACC), the concept of the effective diffusivity has been utilized as proposed by the ref (32), as the effective thermal and hydraulic difficulties have been evaluated according to the following,
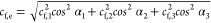
30
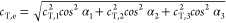
31where *c*_f,e_ and *c*_T,e_ are the effective hydraulic and thermal diffusivity, respectively. the subscripts (1–3) denote the respected quantity along the different axes. In this case, *c*_f,1_ = *c*_f,2_ = *c*_f,∥_, and *c*_f,3_ = *c*_f,⊥_ and the thermal diffusivity is treated in the same way. The angle α is the angle between the gradient and the axes. For the comparison between the results, the axes of the bedding are aligned with the principle axes as in the reference.

For the isotropic case (ICC), only the diffusivity along the bedding planes is considered.

The results demonstrate a strong agreement between the present model and the Laplace inversion method across all the conditions evaluated. This consistency is evident for both isotropic (ICC) and anisotropic (ACC) hydraulic and thermal conductivity scenarios at different time scales (t* = 0.1 and t* = 10), confirming the validity of the current work. However, a noticeable discrepancy arises between the Error function method and the other two methods, particularly at t* = 10. This deviation results from the assumptions made in the Error function method, which are based on early time and small radial distance conditions, as described by the original authors.^32^ The simplifications inherent in this approach limit its accuracy at later times, leading to the observed differences. This comparison presented in the figures confirms both the verification and validation of the present solution.

### 3.2 Stresses Evaluation around the Wellbore

In this section, the present model is applied to analyze the stability of the wellbore using data from Tables 2 and 3, assuming isotropic conditions for the thermal and hydraulic diffusivity. As mentioned earlier, the four time-dependent wellbore stability models are considered to calculate temperature, pressure, stress distributions, and strength reduction when using drilling fluids with varying temperatures and salinity.

**Table 3:** Input Data of the Different Wellbore Stability Models^27^

parameter	value
drilling data	
original pore pressure *P*_o_ (MPa)	10
original formation temperature *T*_o_ (°C)	80
borehole pressure *P*_w_ (MPa)	12
temperature of the cold mud *T*_w1_ (°C)	20
temperature of the hot mud *T*_w2_ (°C)	140
well profile data	
well radius (m)	0.1
well Azimuth from σ_H_ direction β (°)	0
well inclination α (°)	0
chemical data	
water activity of shale, *a*_wsh_	0.84
water activity of low salinity mud (deionized water), *a*_wm1_	1
water activity of high salinity mud (KCOOH), *a*_wm2_	0.4
membrane efficiency, *I*_m_	0.1
partial molar volume of water, *V* (m^3^/g-mole)	1.8 × 10^–5^
gas constant, *R* (kg·m^2^/s^2^·g-mole·°K)	8.314
shale strength reduction by deionized water	25%
shale strength reduction by KCOOH mud	0%

**Figure 5:**
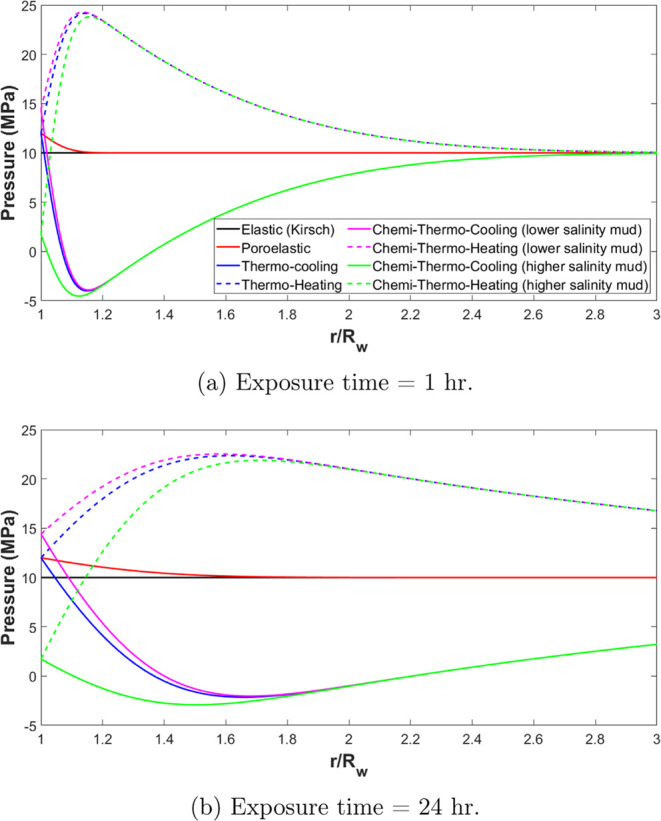
Pressure distribution inside the formation considering various models. (a) Exposure time = 1 hr. (b) Exposure time = 24 hr.

Figure 5a,5b show pressure distribution at different radii from the wellbore using different wellbore stability models after 1 and 24 h from the formation exposure to the drilling fluid, respectively.

A slight increase in the formation pressure with a maximum magnitude at the wellbore walls can be in the observed poroelastic model. This is because of overbalanced drilling conditions, and the fluid diffusion occurs from the wellbore into the formation which leads to an increase in the formation pressure.

By examining the thermal effect, a significant influence on formation pressure is noted. It is observed that after 1 h of exposure to drilling, the formation pressure increases from 10 to 24 MPa when the temperature of the drilling fluid exceeds the formation temperature by 60 °C (Δ*T* = +60 °C). Conversely, when the formation temperature exceeds the drilling fluid temperature, a notable decrease in formation pressure is observed. An increase in temperature causes the expansion of the formation fluid, rock grains, and structure. For a given increase in temperature, the volume change of formation fluid inside the pore spaces is greater than the volume change of porosity, and hence the pore pressure increases. The pressure is then dissipated due to Darcy flow, as can be noticed in Figure 5b. The peak pressure magnitude is decreased and also shifted deep inside the formation. The magnitude and dissipation time needed for that phenomenon depends on the thermal diffusion in comparison with the hydraulic diffusivity of the rock. In the case of low hydraulic diffusivity, and high thermal conductivity as in the case of clay formation, this effect is maximized. This effect has been also noted by previous research.^42,43^

It is worth mentioning that It is acknowledged that a temperature difference of ±60 °C may seem large under conventional drilling conditions. However, such temperature differences are not uncommon in certain drilling environments. For instance, geothermal wells often experience significant temperature gradients, with temperature differences of this magnitude being typical in many geothermal drilling scenarios.^44−46^ Similarly, high-pressure high-temperature (HPHT) wells, as well as deep offshore wells and wells drilled in permafrost regions, can also experience significant temperature differences due to extreme depth, pressure, and environmental conditions.^47^

Finally, noticeable effect on the formation pressure distribution considering the chemical effect. The drilling fluid with lower salinity mud (higher water activity) causes fluid diffusion from the wellbore into the formation by the osmosis pressure which increases the pore pressure, while higher salinity mud reverses the effect. By comparison to the thermal effect, the chemical effect has a shallow influence near the wellbore walls. As can be seen from Figure 5a, the change in the formation pressure after 1 h due to the osmosis only reached 1.2 of the wellbore radius and extended to 1.8 after 24 h.

**Figure 6:**
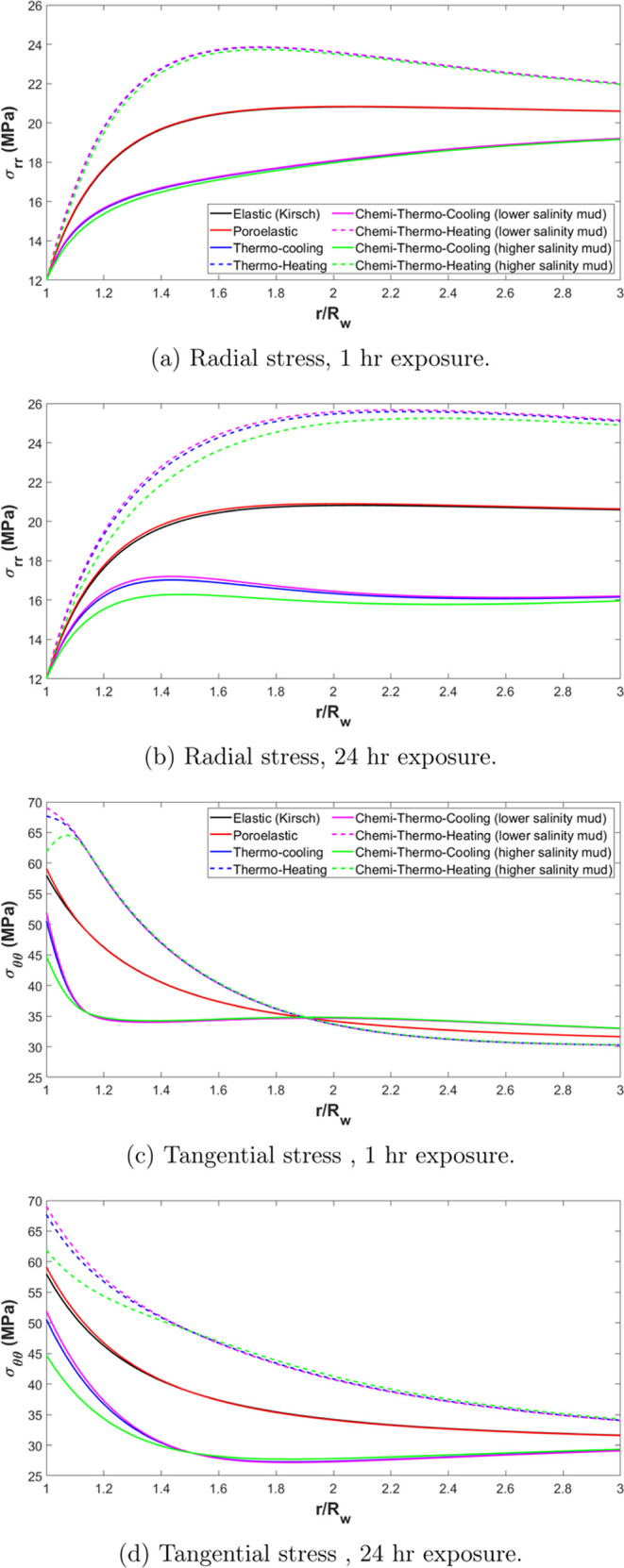
Stress distribution inside the formation considering various models. (a) Radial stress, 1 hr exposure. (b) Radial stress, 24 hr exposure. (c) Tangential stress, 1 hr exposure. (d) Tangential stress, 24 hr exposure.

For wellbore stability analysis, it may maybe more relevant to investigate the resultant stresses acting on the formation, and to further analyze whether this formation will be able to hold the stresses without failure or not. For that reason, a comparison between resultant stresses considering the four main effects is performed as shown in Figure 6. By examining the results in comparison to the base model (elastic), one can observe a small increase in the radial and tangential stresses utilizing the poroelastic model.

Considering the thermal effects, a significant change in both radial and tangential stresses can be observed in comparison to the elastic model for both heating and cooling scenarios. Here, we can differentiate between two effects. First is the stresses resulting from the expansion/contraction of the solid material of the rock due to the temperature difference, including the pore space itself. And second is the stresses resulting from the expansion/contraction of fluid inside the pore space due to the temperature change. To differentiate between both effects, one should analyze Figure 6 in correlation with Figure 5.

Initially, a rapid and shallow change in stresses is observed, which is attributed to the immediate pressure increase within the pore spaces. This is evident in Figure 6a,c, which show the stress distribution after 1 h. At the wellbore wall (*r* = *R*_w_), the tangential stress reaches 68 MPa in the thermoporoelastic model, compared to 58 MPa in the elastic model. For radial stresses, both models show equal stress at the wellbore wall initially. However, deeper inside the formation, at *r* = 1.7 *R*_w_, the thermoporoelastic model records a maximum radial stress of 24 MPa, compared to 20 MPa for the elastic model. Notably, for the thermoporoelastic model, the stresses start to decrease as one moves deeper into the formation, a trend also observed for tangential stresses. However, in Figure 6b,6d, which display the results after 24 h, the stress distribution within the formation becomes more monotonic, with no rapid changes. This gradual change reflects the slower process of thermal expansion/contraction of the rock matrix. In the case of cooling, these phenomena are mirrored, with the stresses decreasing instead. These variations in stress are evident in both radial and tangential stresses.

It is worth noting that after 1 h, the thermoporoelastic model reveals an interesting behavior with the tangential stress observed at a radius greater than 1.9 times the wellbore radius (*r* > 1.9 *R*_w_) where the effect of heating is minimal, as demonstrated in Figure 6c. One explanation for that result is at distances further away from the wellbore, the expansion effects are more pronounced in the surrounding formation, where the pressure buildup has dissipated, leading to a localized reduction in stress, which creates a zone of minimum stress deeper in the formation.

The chemical interactions between the fluid and the rock formation also show a role in stress distribution when considering fluids of varying salinity. In comparison to the thermal effect, the chemical effect is much slower, however, it can still influence both radial and tangential stresses over time. For radial stresses, after 1 h as in Figure 6a, the high salinity fluid causes a slight decrease in the near wellbore area (*r* = 1.7 *R*_w_). Beyond this point, the radial stresses align with those predicted by the thermoporoelastic model, and the chemical effect vanishes. This slight decrease in radial stress can be attributed to the osmotic pressure differences and ion exchange processes, which slightly alter the stress distribution close to the wellbore. On the other hand, for low salinity fluid, the radial stress distribution closely follows the thermoporoelastic model, making it difficult to distinguish any significant difference between the chemi-thermoporoelastic and thermoporoelastic models at this stage. However, after 24 h, the chemical effect becomes more pronounced. In the high salinity case, the influence on radial stresses extends deeper into the formation, reaching a radius of approximately 3 *R*_w_. The more significant effect observed in the high salinity case after 24 h is likely due to the prolonged interaction between the ions in the fluid and the rock matrix, which leads to more changes in pore pressure and stress redistribution over time. Concerning the tangential stresses, the impact of the chemical effects acts differently. After 1 h, the change in tangential stresses due to chemical effects is limited to a radius very shallow to the wellbore walls (*r* = 1.15 *R*_w_). However, as time progresses, the tangential stresses are further influenced by the ongoing chemical reactions, with the effect reaching a radius of about 1.5 *R*_w_ after 24 h. This gradual expansion of the affected zone indicates a slow but steady redistribution of tangential stresses as the chemical interactions progress.

### 3.3 Model Application for Wellbore Stability Analysis

To translate the discussion of the previous section into practical applications within the drilling industry, it is essential to analyze the predicted stability and identify the stable regions around the wellbore. As elaborated in the previous section, the complex interactions between thermal, chemical, and poroelastic effects significantly influence the stress distribution, which in turn affects wellbore stability. Each factor contributes individually to stability conditions. Thermal effects primarily induce stress through the expansion or contraction of both rock and pore fluid, while chemical interactions, driven by osmotic pressures and ion exchanges, gradually alter stress close to the wellbore. These thermal and chemical effects combine with poroelastic responses to fluid diffusion under overbalanced drilling conditions, leading to intricate stress redistribution patterns that influence both radial and tangential stresses over time.

Another key factor in this analysis is the failure criterion, which determines the conditions under which the rock surrounding the wellbore may fail or remain stable.

**Figure 7:**
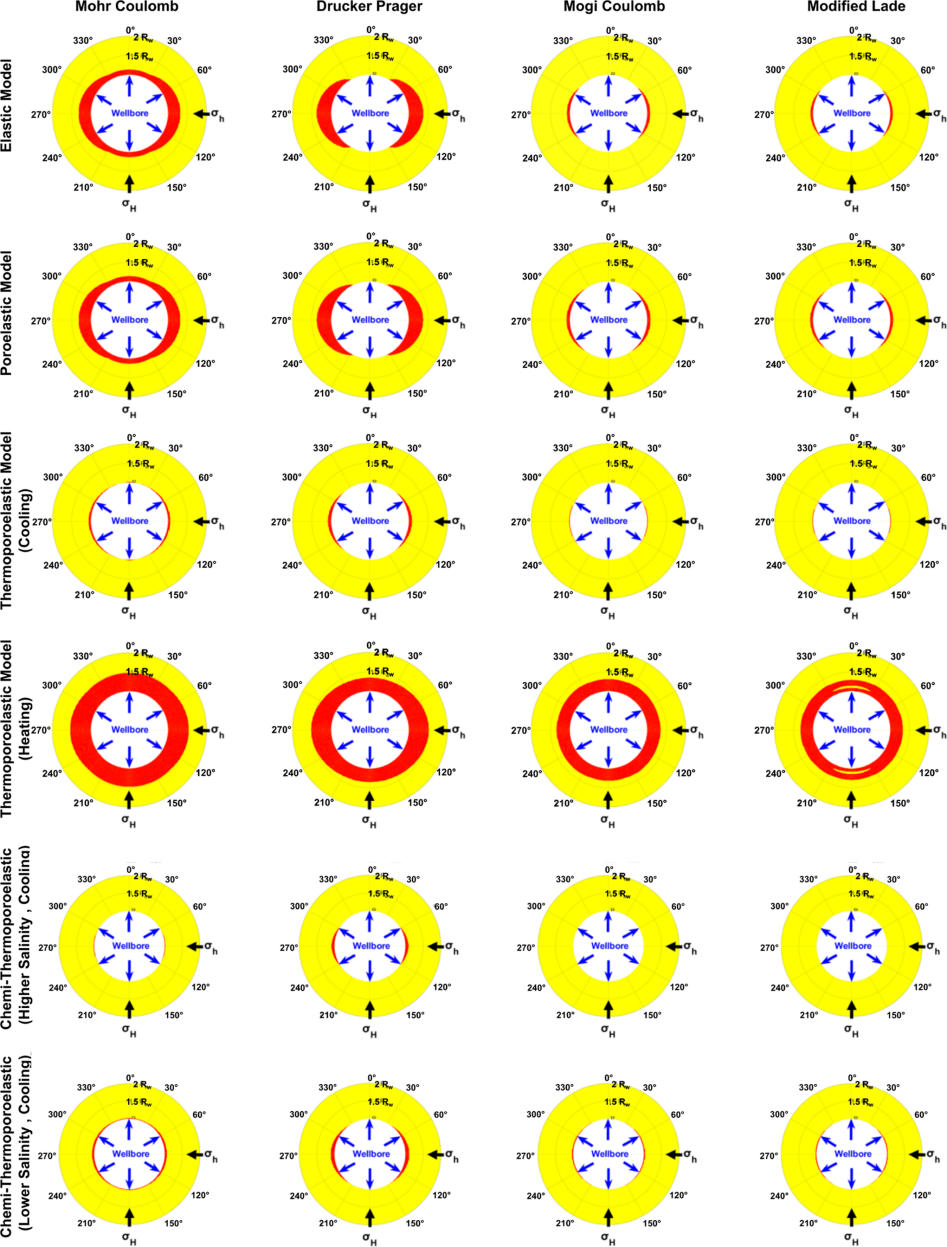
Wellbore collapse prediction using different failure criteria and stress models.

Figure 7 shows the predicted collapse failure zone around the borehole using different modeling scenarios around a wellbore. Each plot corresponds to a specific combination of failure criterion and model conditions, as labeled at the top of each plot. The figure is organized as rows and columns. Each row presents different types of models or conditions, and the columns represent the different failure criteria. All figures are generated using the 1 h stress result.

From the figure, one can observe that Mohr-Coulomb and Drucker-Prager failure criteria generally show more extensive yielding zones than Mogi-Coulomb and Modified-Lade criteria. The failure criterion significantly impacts the predicted failure zones. Mohr-Coulomb and Drucker-Prager show similar patterns, while Mogi-Coulomb and Modified-Lade criteria exhibit unique stress distribution characteristics. As can be seen, Mohr-Coulomb displays a more uniform yielded zone around the wellbore, indicating broader concentrated yielding occurring at azimuths perpendicular to the maximum horizontal stress. This pattern implies that the Mohr-Coulomb criterion may be more sensitive to the uniformity of applied stresses and may predict washout around the entire borehole wall. Drucker-Prager, on the other hand, is also extensive but slightly more localized than Mohr-Coulomb, and like Mohr-Coulomb, predicts broader yielding areas.

In contrast, Mogi-Coulomb and Modified-Lade predict more localized yielding patterns, indicating stress concentration primarily in the direction of the minimum principal stress. This results in breakout orientations rather than a washout pattern, as seen in the Mohr-Coulomb case. Mogi-Coulomb and Modified-Lade thus suggest a more anisotropic stress distribution, where stress changes are less pronounced across the wellbore, reducing the likelihood of complete wellbore wall failure. This characteristic implies that these criteria are more sensitive to triaxial stress conditions and predict a gradual initiation of failure that localizes rather than disperses stress, as reflected in the smaller, distinct yielded zones shown in the analysis.

The Poroelastic model shows a similar pattern as in the elastic model when it comes to failure zone prediction. Mohr-Coulomb and Drucker-Prager show more conservative behavior, predicting larger yielded zones, while Modified-Lade is less conservative.

Thermal effects show a noticeable influence on the predicted yielded zones across all failure criteria utilized in this study. First, cooling induces contraction, generally reducing stress levels, but potentially increasing tensile stresses around the wellbore. Mohr-Coulomb and Drucker-Prager criteria continue to predict failure occurrence, with a noticeable reduction compared to the nonthermal poro-elastic model. On the other hand, heating shows exacerbates plastic deformation, increasing the risk of wellbore instability. The difference between failure criteria becomes a little less distinguishable, as they all predict the same failure pattern. However, Mohr-Coulomb and Drucker-Prager showed the most significant increases in the yielded zone.

Finally, combined chemical and thermal effects, with higher and lower salinity and thermal changes using the chemi-thermoporoelastic model. Higher salinity enhances the stability of the formation around the wellbore with the four failure criteria. In comparison with the thermo-poro-elastic model, under cooling conditions, one can notice that there is no failure predicted in the case of Mogi-Coulomb or Modified-Lade. In the lower salinity case, there is no noticeable difference compared to the thermoporoelastic scenario. The predicted failure zone is almost identical, with only a slight change observed as a small expansion of the yielded zone around the circumference of the wellbore, rather than deeper within the formation.

Based on these observations, we conclude that Mohr-Coulomb and Drucker-Prager criteria provide more conservative predictions, making them suitable for applications in high-uncertainty or less-developed fields where stability is critical and a larger safety margin is preferred. These criteria are valuable in situations where limited data are available, as they require only a few key parameters to be utilized, such as cohesion and friction angle. This makes them advantageous in early stage field development, where detailed geomechanical data may be lacking.

In contrast, the Mogi-Coulomb and Modified-Lade criteria, which predict smaller yielded zones, may be more suitable for well-characterized, more-developed fields with lower uncertainty and greater operational knowledge. Therefore, the choice of failure criterion should be guided by both the specific stability requirements of the drilling operation and the available field data.

These results show that the choice of failure criterion significantly impacts the predicted stress distribution and extent of plastic deformation around the wellbore. Mohr-Coulomb and Drucker-Prager are more conservative, predicting larger plastic zones, while Mogi-Coulomb and Modified-Lade are less conservative, with Modified-Lade often predicting the least deformation. The interaction of thermal and chemical effects with the selected failure criterion can significantly alter the predicted wellbore stability. Heating generally increases the extent of plastic deformation. Cooling tends to reduce the plastic zones, but the overall pattern remains influenced by the failure criterion.

### 3.4 Application of Wellbore Stability Analysis for Predicting Safe Mud Weight Margins

As an application of this study, wellbore stability analysis is employed to predict safe mud weight margins, for preventing wellbore collapse and fracturing. It is defined by the difference between the maximum allowable mud weight (to prevent fracture of the formation) and the minimum allowable mud weight (to prevent wellbore collapse). This difference, *P*_window_ = *P*_frac_ – *P*_collapse_ defines the safe operational zone. The larger *P*_window_ magnitudes correspond to a wider mud window with less risk for instability issue, *P*_window_ ≤ 0 corresponds to a nonstable wellbore.

**Figure 8:**
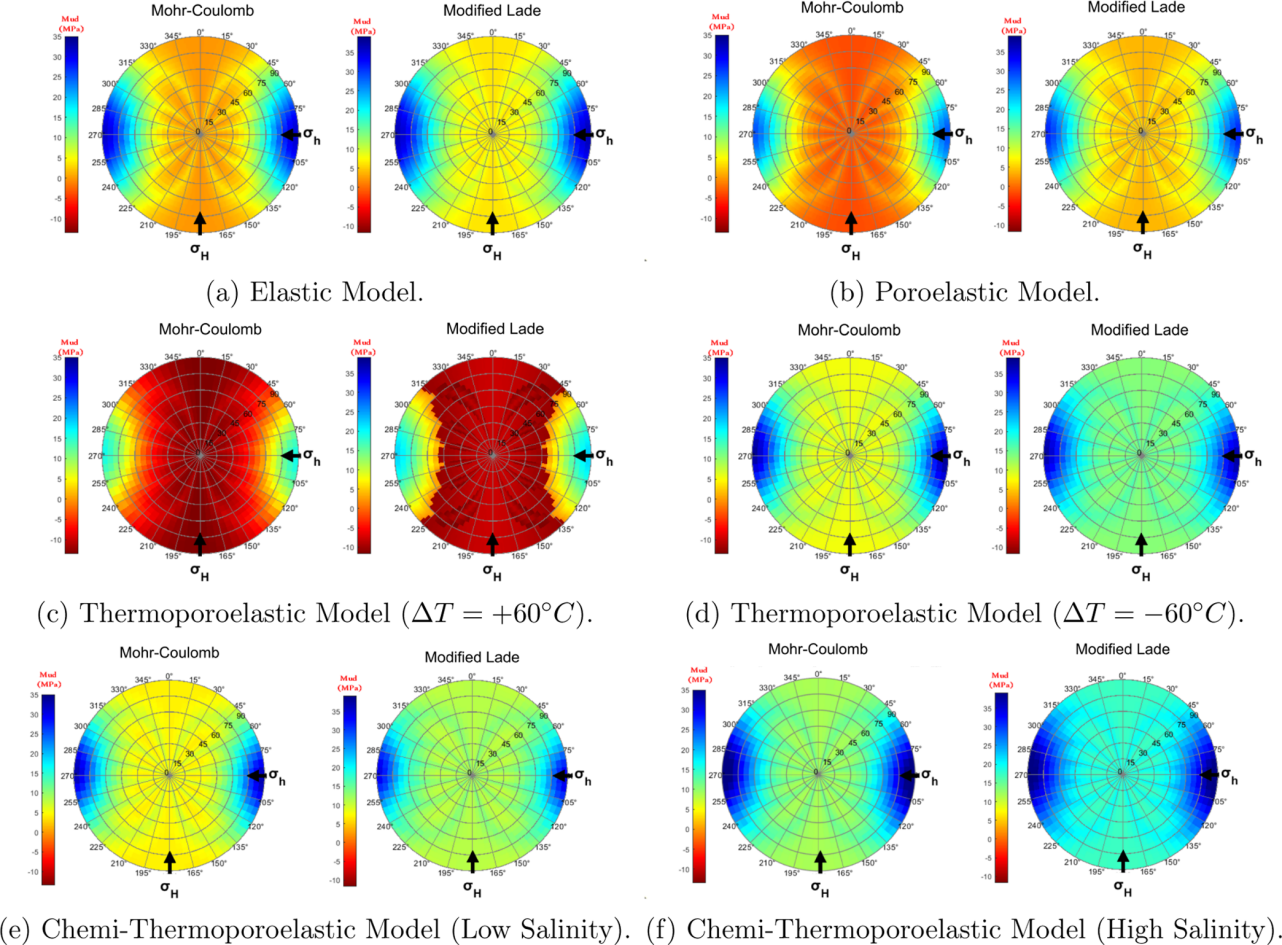
Mud window prediction using different stress calculation models and collapse failure criteria (σ_*V*_ = 30, σ_*H*_ = 30, σ_*h*_ = 20 MPa) at *r*/*r*_w_ = 1, *t* = 1 min.

Wellbore orientation (inclination angle and direction) can alter the resulting stresses acting on the wellbore walls, which can influence the stability of the well. Therefore, in this analysis, different models and failure criteria are used to predict the mud window across various wellbore orientations and inclinations. The results are visualized using stereo net plots as shown in Figure 8, where Azimuth (0–360° around the circumference) represents the orientation of the wellbore in the horizontal plane, with North at 0° and South at 180°, and inclination is represented radially, with vertical wellbores at the center (0°) and increasing inclination toward the outer edges (90°). Mohr-Coulomb and Modified Lade collapse failure criteria were selected for this analysis, and they represent the most and the least conservative criteria as described in the previous section. A MATLAB code is developed for calculating the minimum and the maximum allowable mud weight for each azimuth and inclination angle as described in Figure 3.

In the elastic model case, the Mohr-Coulomb criterion predicts a critically narrow mud window, particularly for vertical wells, and horizontal wells in the direction of the maximum horizontal stress. However, for high-angle wells, in the direction of the minimum horizontal stress the mud window is wider. When the Modified Lade criterion is applied to the elastic model, the mud window becomes wider, reflecting a less conservative estimate of wellbore stability.

As pore pressure and thermal effects are considered (in the poroelastic and thermoporoelastic models), the mud window narrows noticeably in case of heating. The influence of temperature is particularly significant. Under high-temperature conditions, the mud window shrinks significantly, with most well orientations, showing red zones, suggesting instability. An increase in formation temperature contributes to increased pore pressures and radial stress expansion, as well as an increase in tangential stress distribution near the wellbore. This combined stress effect can cause reduced formation integrity, especially in zones prone to thermal expansion, resulting in a narrower mud-weight window. Lower temperatures (−60 °C) somewhat alleviate this issue. The inclusion of chemical effects shows minor effects on the stable mud window.

It is worth noting that the anisotropic stress state of the formation, defined by the stress magnitudes σ_*V*_ = 30 MPa, σ_*H*_ = 30 MPa, σ_*h*_ = 20 MPa, has a significant effect on wellbore stability. Due to this stress anisotropy, horizontal wells oriented in the direction of the minimum horizontal stress (σ_*h*_) show the most stable conditions, as evidenced by larger blue zones. This occurs because, in these orientations, the differential stresses acting on the wellbore are smaller, which reduces the likelihood of collapse or fracture. In contrast, wells aligned with the maximum horizontal stress (σ_*H*_) are less stable, which is reflected by red zones (negative mud window values) indicating unsafe drilling conditions. Since the vertical stress (σ_*V*_) is equal to the maximum horizontal stress (σ_*H*_), this results in higher stress concentrations around the wellbore and narrows the mud window.

In the elastic and poroelastic models, the failure criterion plays an important role in determining the size of the mud window and the regions of stability. In the thermoporoelastic models, which account for temperature effects, both the Mohr-Coulomb and Modified Lade criteria yield very similar results. The convergence of the two criteria under thermal effects suggests that thermal stresses dominate the failure mechanisms, reducing the differences between the two criteria. As a result, both models predict similar regions of stability.

### 3.5 Effect of Time on the Collapse Area and Mud Window

**Figure 9:**
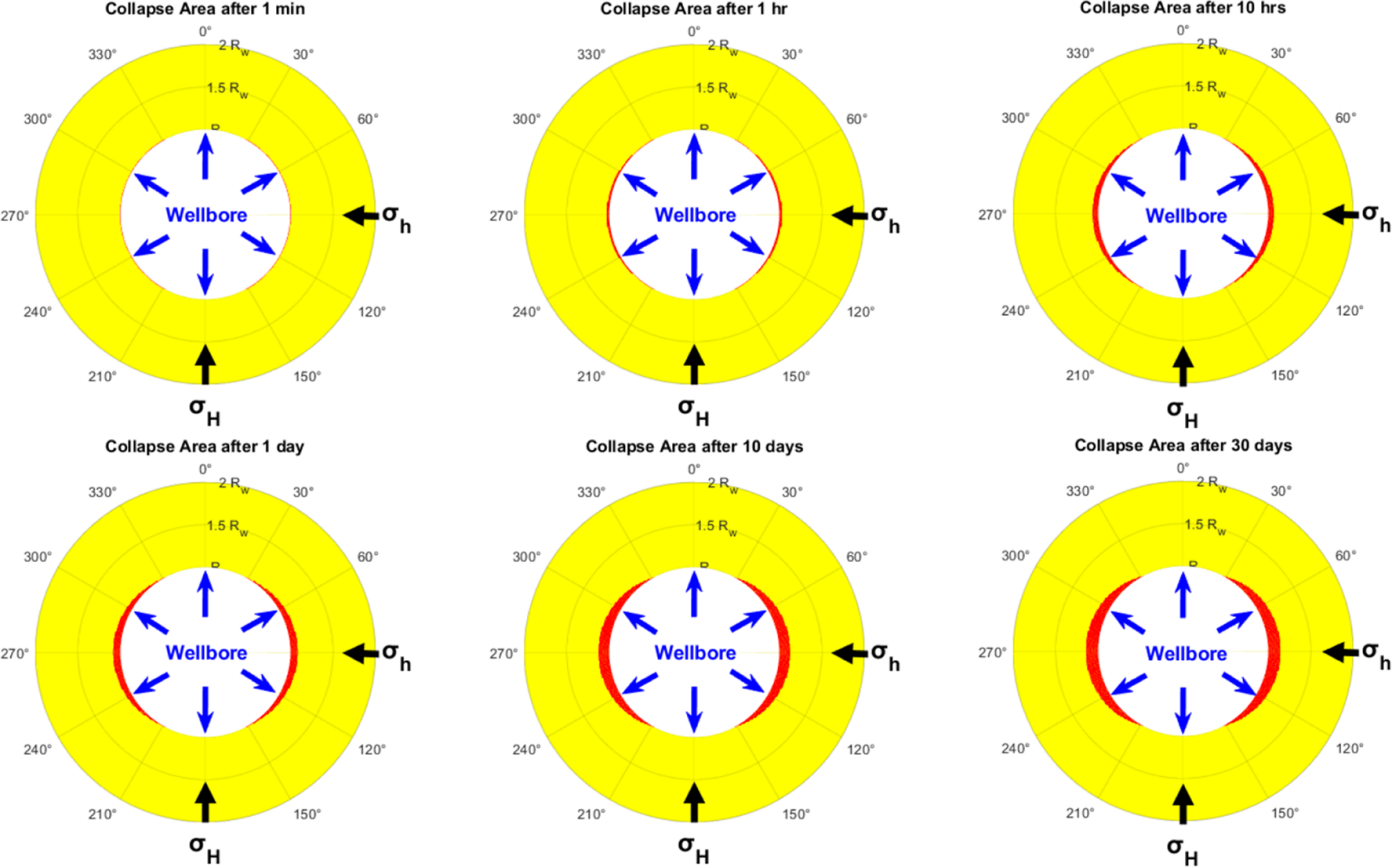
Collapse area using the chemi-thermoporoelastic model and Mogi-Coulomb failure criterion in case of cold-low salinity mud at different times.

Further efforts to demonstrate the effect of time on the stability of the wellbore, the collapse area has been predicted at different times using the chemi-thermoporoelastic model and Mogi-Coulomb failure criterion in the case of cold-low salinity mud. Figure 9 shows that the collapse area increases with time since the cooling effect reduces the tangential stress and the pore pressure near the wellbore diminishes with time as shown in Figure 4 and Figure 6c,6d after 1 and 24 h. Also, more fluid diffuses from the wellbore into the formation by the poroelastic and chemical effect which increases the pore pressure and the tangential stress making the formation more susceptible to collapse. This diffusion is driven by the overbalanced drilling conditions, which cause a differential pressure between the wellbore and the surrounding formation that drives the fluid from the wellbore into the formation. This fluid diffusion increases the pore pressure in the formation and results in an elevated tangential stress near the wellbore. The chemical effect, on the other hand, further enhances fluid diffusion through osmosis. The osmotic pressure difference due to different fluid salinity between the drilling fluid in the wellbore and the formation fluid drives additional fluid flow into the formation.

**Figure 10:**
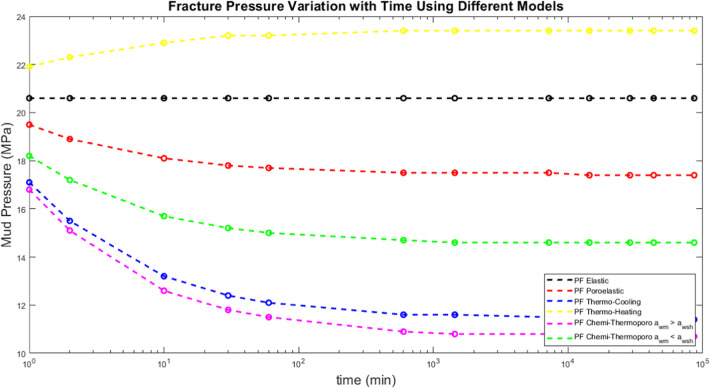
Fracture pressure variation with time using different stability models.

The effect of time on the fracture pressure has been presented using the different models and Mogi-Coulomb failure criterion as shown in Figure 10. The elastic model does not consider any time-dependent effects, so the fracture pressure is constant with time. In the other models, fracture pressure changes with time until some point (*t* = 1 h) when it starts to stabilize. Starting with the poroelastic effect, as time passes, it causes more fluid diffusion inside the formation which increases the pore pressure and decreases the effective stress making the formation more susceptible to fracture so the fracture pressure decreases with time. As the thermal effect on pressure decreases with time near the wellbore, the pore pressure increases with time during cooling conditions (Δ*T* = −60 °C) and decreases the minimum effective principal stress making the formation fracture easier. So, the fracture pressure decreases with time. However, during heating conditions (Δ*T* = +60 °C), the pore pressure decreases with time which increases the minimum effective principal stress making the formation more stable against tensile fracturing. Considering the chemical effect, we can note an increase in the difference between the fracture pressure predicted by the chemi-thermoporoelastic model and the thermoporoelastic model during both higher and lower salinity mud. This means that the chemical effect increases with time increasing the fracture pressure during higher salinity mud (*a*_wm_ < *a*_wsh_) and decreasing it during lower salinity mud (*a*_wm_ > *a*_wsh_). As time passes, low water activity mud causes more fluid diffusion from the formation to the wellbore by osmosis effect which decreases the pore pressure and increases the minimum effective principal stress making the rock more stable against fracturing.

**Figure 11:**
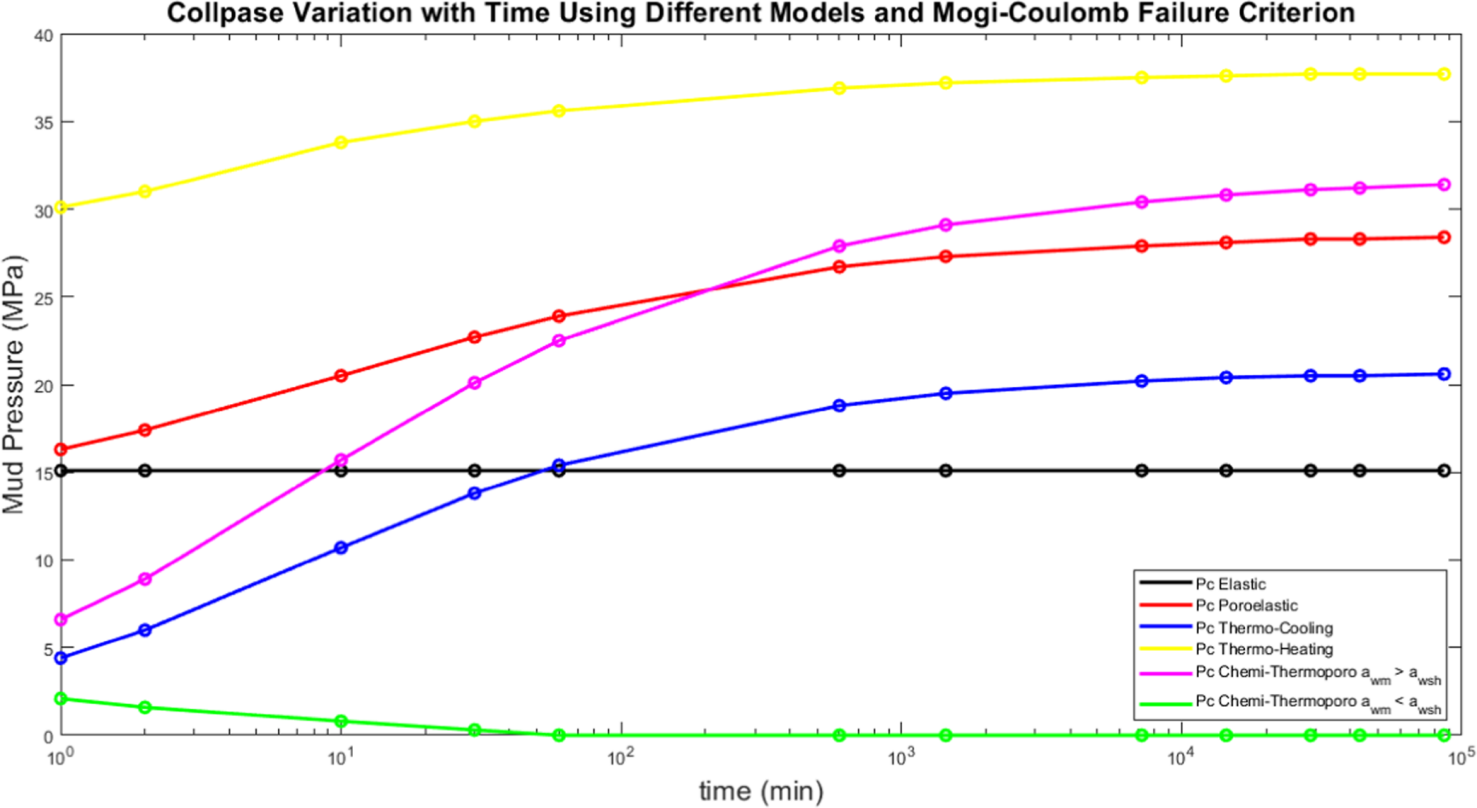
Collapse pressure variation with time using different stability models and Mogi-Coulomb failure criterion.

Figure 11 shows the effect of time on the collapse pressure using different models and Mogi-Coulomb failure criterion. In comparison with fracture pressure variation, the collapse pressure takes more time to stabilize (*t* = 1 day). Also, the thermal effect worsens the formation’s stability to collapse with time increasing the collapse pressure during both cooling and heating the formation. Moving to the chemical effect, using higher salinity (*a*_wm_ < *a*_wsh_) mud enhances the stability to collapse and decreases the collapse pressure with time, while lower salinity mud has a reverse effect.

**Figure 12:**
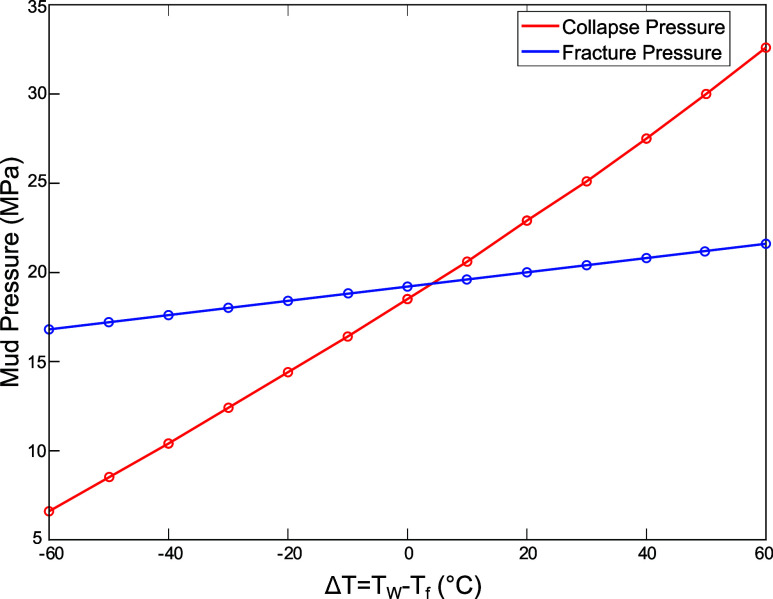
Collapse pressure and fracture pressure calculated according to chemi-thermoporoelastic model, and Mogi-Coulomb failure criterion rate *t* = 1 min and various differential temperatures.

Furthermore, to provide a more comprehensive analysis regarding the thermal effects, additional simulations with a temperature difference range, extending from +60 to −60 °C were performed as shown in Figure 12.

The figure shows the relationship between collapse pressure and fracture pressure and the temperature difference (Δ*T*), which is calculated as the difference between the wellbore temperature (*T*_w_) and the formation temperature (*T*_f_). The collapse and fracture pressure were calculated using the Chemo-Thermoporoelastic model and the Mogi-Coulomb Failure Criterion at *t* = 1 min. It is noticeable that The width of the mud window narrows significantly as Δ*T* increases. As the temperature difference (Δ*T*) increases in the heating scenario, both the collapse pressure—representing the minimum mud weight required to prevent wellbore collapse—and the fracture pressure—indicating the maximum permissible mud weight to avoid formation fracturing—show an upward trend. However, when the temperature difference Δ*T* ≥ + 5 °C, the collapse pressure begins to exceed the fracture pressure, resulting in a negative mud window. This indicates that maintaining a stable wellbore becomes unachievable under such differential temperature conditions, as no viable mud weight range can simultaneously satisfy both stability criteria.

Conversely, unlike the heating scenario (+Δ*T*), in the cooling scenario, the collapse pressure consistently remains lower than the fracture pressure across the range of negative temperature differences. Additionally, it can be observed that the mud window widens as the temperature of the drilling fluid decreases relative to the formation temperature.

## 4 Conclusions

This work introduced a novel holistic approach to wellbore stability analysis by integrating poroelastic, thermal, and chemical effects into a comprehensive modeling framework. An in-depth examination of the coupled interactions between these effects was provided, yielding new findings into stress distribution and instability risk in high-pressure, high-temperature environments. Four stability models were used to analyze wellbore stability, comparing four shear failure criteria—Mohr-Coulomb, Drucker-Prager, Mogi-Coulomb, and Modified-Lade—to predict collapse areas, safe mud windows, and optimal wellbore trajectories. The study highlighted the significant role of time-dependent effects such as hydraulic, thermal, and chemical interactions, as well as drilling conditions such as mud pressure, temperature, salinity, and wellbore trajectory, on stability analysis. The results show that, due to the high thermal diffusivity of shale, thermal effects have a more pronounced impact on wellbore stability compared to poroelastic and chemical effects. The poroelastic effect increases the collapse area by 5%, while the thermal effect minimizes the collapse area by 80% during formation cooling and enlarges it by 140% during formation heating. The chemical effect decreases the collapse area by 20% using higher salinity mud and increases it by 10% using lower salinity mud. Regarding fracture pressure, the hydraulic effect reduces the fracture pressure from 20.4 to 17.4 MPa, a decrease of 15%. The thermal effect decreases fracture pressure by 30% during formation cooling and increases it by 15% during heating. Higher salinity mud enhances fracture stability by increasing fracture pressure by 15%, whereas lower salinity mud decreases it by 7%. Additionally, the anisotropic stress state of the formation significantly impacts wellbore stability, with a larger collapse area observed in the direction of the minimum principal stress. The comparison of four rock failure criteria is a unique contribution of this paper. Mohr-Coulomb and Drucker-Prager, which predicted 15–20% larger collapse areas, provide a more conservative approach. The Mohr-Coulomb criterion predicts a critical narrow mud window for vertical wells and horizontal wells oriented in the direction of the maximum horizontal stress, while for high-angle wells oriented in the direction of the minimum horizontal stress, the mud window is wider. The Modified Lade criterion reflects a less conservative estimate with a wider mud window. To enhance the understanding of wellbore stability and identify key influencing parameters, a global sensitivity analysis using Monte Carlo simulation could be employed, providing valuable insights for robust and resilient wellbore design.
